# *Chaetocarpus castanocarpus* Bark Extract Alleviates Pain, Pyrexia, Diarrhea, Hyperglycemia, and Oxidative Stress: Evidence From Integrated In Vitro and In Vivo Studies

**DOI:** 10.1002/fsn3.72221

**Published:** 2026-08-16

**Authors:** Rakibur Rahman, Farjana Akter Nipa, Taimun Siddique, Tania Rahman, Abed Foyez Rafi, Alfesani, Ziaul Karim, Tuhin Chandra Das, Riniara Khatun, Abdur Rauf, Hassan A. Hemeg

**Affiliations:** ^1^ Department of Pharmacy, Faculty of Science and Engineering International Islamic University Chittagong Chattogram Bangladesh; ^2^ Department of Chemistry University of Swabi Swabi Pakistan; ^3^ Department of Clinical Laboratory Sciences College of Applied Medical Sciences Riyadh Saudi Arabia

**Keywords:** antidiarrheal, antinociceptive, antioxidant, antipyretic, *Chaetocarpus castanocarpus*, hypoglycemic

## Abstract

There is a history of using the medicinal plant *Chaetocarpus* genus, native to Southeast Asia, to treat a wide range of illnesses. The absence of detailed pharmacological validation for this plant is unexpected, considering its importance in ethnopharmacology. This study was supposed to conduct a laboratory evaluation of the pharmacological properties of the methanol extract of *Chaetocarpus castanocarpus* bark (MECCB). The MECCB was prepared by cold maceration and then subjected to phytochemical measurement (total phenolic, flavonoid, and tannin content) and antioxidant testing ([2,2‐diphenyl‐1‐picrylhydrazyl (DPPH) radical scavenging]). Oral glucose tolerance tests were utilized to evaluate hypoglycemic potential, yeast‐induced pyrexia to measure antipyretic effects, castor oil‐induced diarrhea to measure antidiarrheal activity, and acetic acid‐induced writhing and formalin tests to evaluate analgesic effects in vivo. The study used standard treatments, 200 and 400 mg/kg MECCB in contrast to the control group in *Swiss albino* mice. At 400 mg/kg, MECCB exhibited a writhing inhibition of 73.86%, demonstrating dose‐dependent antinociceptive effectiveness. MECCB‐treated groups showed a significant reduction in fever compared to the vehicle control group. A decrease of 37.06% in intestinal motility and a reduction of 58.53% in diarrheal feces were among the antidiarrheal effects. The fast drop in glucose levels (Δ = 6.5 mmol/L) caused by hypoglycemia was comparable to the effects of glibenclamide. The IC50 of MECCB in the DPPH assay is 418.73 μg/mL, demonstrating its antioxidant activity. This activity is associated with the high amounts of flavonoids (280.143 ± 45.122 mg QE/g) and phenolics (364.917 ± 27.0159 mg GAE/g) present. MECCB's potential as a natural medication is supported by its positive effects on pain relief, fever reduction, diarrhea treatment, and glucose regulation. Additional research is needed to identify active compounds and elucidate their mechanisms.

## Graphical abstract

Graphical abstract illustrating *Chaetocarpus castanocarpus* bark extract alleviates pain, pyrexia, diarrhea, hyperglycemia, and oxidative stress: Evidence from integrated in vitro and in vivo studies.

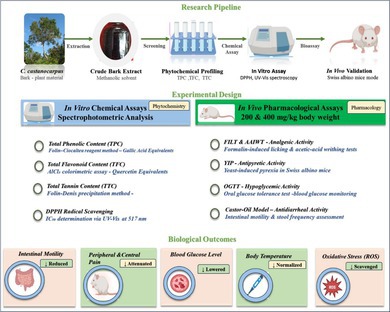

## 1 Introduction

Natural products have long been applied by humans for therapeutic purposes. In the 19th century, as chemistry developed, more research was conducted on plants to discover whether they could be used for medicinal purposes. There are medical uses for the phytochemicals used in herbal or pharmaceutical drugs (Latif and Nawaz 2025; Wal et al. 2025). The active components found in herbs have pharmacological effects. Herbal remedies are less potent than synthetic medications, yet they are less expensive and have fewer side effects than synthetic treatments. All medicines, whether natural or artificial, must meet specific standards to be considered safe; they must be non‐harmful, practical, specific, stable, and potent (Bursal et al. 2019; Chaachouay and Zidane 2024).

Locals in Bangladesh refer to *Chaetocarpus castanocarpus* (Roxb.) as Muttul jam, and it is from the Euphorbiaceae family (Prado 2013). This particular plant is indigenous to the countries of Bangladesh, Myanmar, Indonesia, India, Cambodia, Sri Lanka, Thailand, Laos, and Vietnam. Naturally growing in the upland areas of Bangladesh, Chittagong and Sylhet are the primary locations for this plant (Prado 2013). Phytochemical analysis has revealed the presence of many phytochemicals, including alkaloids, flavonoids, glycosides, terpenoids, coumarins, and tannins. Additionally, this plant offers potential medicinal benefits that can be used for the management of neurological disorders (Dey and Rahman 2013; Rahman et al. 2024).

The chemical process of oxidation allows an oxidizing agent to acquire an electron or a hydrogen atom from a material. “Free radicals” are byproducts of oxidation reactions. Thus, these radicals have the ability to set off chain reactions that, when carried out within a cell, might cause harm or perhaps cell death (Boarescu et al. 2023). The antioxidant defense mechanisms of an organism are responsible for stopping these chain events by eliminating free radical intermediates and blocking other oxidative processes. Degenerative illnesses caused by oxidative stress, such as cancer, Parkinson's, Alzheimer's, and atherosclerosis, can be prevented through the consumption of plant‐derived antioxidants (Chandimali et al. 2025; Reza et al. 2021).

The management of chronic pain is a significant therapeutic challenge due to the severe side effects of current analgesics, such as non‐steroidal anti‐inflammatory drugs (NSAIDs) and opioids. Selective cyclooxygenase‐2 (COX‐2) inhibitors have fewer gastrointestinal (GI) side effects, but they still pose a risk to the cardiovascular system, and opioids can lead to tolerance, addiction, and respiratory depression (Yimer et al. 2020). Only 30% of patients have a 50% reduction in pain with current medications, which frequently results in insufficient pain relief. Alternative treatments are desperately needed in light of these drawbacks and the impact that untreated pain has on society (Subedi et al. 2016). Medicinal plants present viable solutions due to their traditional use, broad accessibility, and lower incidence of side effects. Plants can provide safer anti‐inflammatory and analgesic compounds, as they are the source of approximately 25% of today's pharmaceuticals (Nunes et al. 2020).

Fever is defined as a rise in body temperature above homeostatic values. The first unusually high body temperature is caused by an imbalance between heat generation and heat loss. The main cause is the hypothalamic “thermostat” mechanism resetting at a higher level. Fevers are complex physiological reactions that can be triggered by non‐infectious conditions, drugs, or malignancies (Emon, Alam, Rudra, Haidar, et al. 2021; Emon, Alam, Rudra, Riya, et al. 2021). Indomethacin is a commonly used medication that blocks the production of prostaglandins by inhibiting the activity of COX enzymes. Prostaglandins are important mediators of inflammation, fever, and pain (Shukla and Mehta 2015). Numerous problems, including gastrointestinal side effects, renal and liver dysfunction, and others, have been associated with currently available non‐steroidal anti‐inflammatory medicines. Therefore, studies aimed at developing novel, safer, and more cost‐effective antipyretic drugs, preferably derived from natural sources, are strongly encouraged (Emon, Alam, Rudra, Haidar, et al. 2021; Emon, Alam, Rudra, Riya, et al. 2021).

In underdeveloped countries, diarrheal infections cause a disproportionately high number of deaths among children (Hartman et al. 2023). Herbal remedies have shown tremendous potential in the treatment of diarrhea using anti‐spasmodic action, delayed intestinal transit, and improved electrolyte reabsorption, among other mechanisms (Damtie 2023). The main components that cause these medicinal benefits are bioactive substances like flavonoids and tannins. There is still a need for extensive pharmacological validation of plants that have been traditionally used to treat diarrhea, such as *Moringa oleifera* and Calpurnia aurea (Belayneh et al. 2024).

Diabetes mellitus is a metabolic condition in which elevated blood sugar levels over an extended period cause symptoms, including frequent urination, increased thirst, and increased hunger. The International Diabetes Federation estimates that 382 million individuals worldwide have diabetes mellitus (Akter et al. 2023). While medication will be necessary for long‐term diabetes management, lifestyle changes can help improve glycemic control in the short term (Ojo et al. 2023). The standard treatment was Glibenclamide, which promotes insulin release by blocking ATP‐sensitive potassium channels found on beta cells, thereby depolarizing the cells and increasing insulin output (Ahangarpour et al. 2019).

Plant‐derived therapies are becoming increasingly important in treating global health issues such as diabetes, helminthiasis, pain, fever, and illnesses associated with oxidative stress, according to recent studies (Belayneh et al. 2024; Subedi et al. 2016). *Chaetocarpus castanocarpus*, containing bioactive compounds such as flavonoids and tannins, presents a promising candidate; however, its pharmacological potential is still underexplored (Dey and Rahman 2013; Rahman et al. 2024). This study evaluates the methanol extract of *C. castanocarpus* bark (MECCB) using in vivo and in vitro methods, focusing on its antidiabetic, analgesic, antipyretic, antidiarrheal, and antioxidant properties to validate its traditional uses and therapeutic potential.

## 2 Methodology

### 2.1 Drugs and Chemicals

All reagents and chemicals employed in this investigation were of analytical standard. Methanol (Sigma Chemical Company, USA) was utilized as a solvent. The pharmaceutical standards that were used were as follows: diclofenac sodium (produced by ACME Laboratories Ltd. in Bangladesh), indomethacin (produced by Aristo Pharma Ltd. in Bangladesh), glibenclamide (produced by Square Pharmaceuticals Ltd. in Bangladesh), and loperamide (produced by Square Pharmaceuticals Ltd. in Bangladesh). To conduct biological tests, formalin, castor oil (provided by WELL's Health Care in Spain), acetic acid (provided by Sigma‐Aldrich in Germany), and Brewer's yeast (provided by Angel Yeast Co. in China) were utilized. Vehicle solutions were made with Tween 80 (BDH Chemicals, UK) and 0.9% NaCl (Social Marketing Company Ltd., Bangladesh). A saline solution containing 1% Tween 80 was used to suspend the methanolic extract of *C. castanocarpus* bark (MECCB), while reference medications were dissolved in isotonic saline (0.9% NaCl) immediately before use.

### 2.2 Extract Preparation

The MECCB was produced, as reported in our earlier work (Rahman et al. 2024). The bark was collected from Kaptai, Bangladesh, and it was authenticated (Voucher No. 201022‐157; verified by Prof. Dr. Shaikh Bakhtear Uddin, University of Chittagong, and R. Khatun, IIUC). After the material was cleaned and allowed to dry in the shade at a temperature of 25°C ± 1°C, it was then powdered into particles measuring 1 mm and extracted using cold maceration (400 g powder in 1:5 w/v methanol, 120 h, 25°C ± 2°C). After being filtered using a Whatman #1 filter, condensed by an evaporator, and stored at 4°C, the extract yielded 3.2% (Ansari et al. 2012).

### 2.3 Experimental Animals

*Swiss albino* mice were obtained from Rajshahi, Bangladesh. Male and female *Swiss albino* mice (*n* = 3/group) were used at random. Data from both sexes were combined as preliminary analysis revealed no significant sex‐related variations in outcomes. These mice were between 5 and 6 weeks old and weighed between 22 and 30 g. The animals were housed in a controlled environment with a 12‐h light/dark cycle, where the temperature was maintained at 23°C ± 2°C and the relative humidity was kept at 55%–60%. A standard pellet meal and filtered water were delivered on demand. After a 48‐h acclimation period, all studies were performed in the International Islamic University Chittagong Department of Pharmacy animal facility.

### 2.4 Animal Grouping and Dosing

The doses of 200 and 400 mg/kg per os (oral administration) (p.o.) were chosen based on a recently published study conducted (Rittika et al. 2025), which confirmed that the 200 and 400 mg/kg doses of plant extract are safe, with no mortality or behavioral abnormalities observed. The control group received the vehicle alone (10 mL/kg), while the doses were given orally (p.o.) as a suspension in 1% Tween‐80. Standard reference medications suitable for each pharmacological test were given to the positive control groups. The standard therapies were protocol‐specific and included the following: diclofenac sodium (10 mg/kg) for analgesia, indomethacin (4 mg/kg) for antipyresis, loperamide (5 mg/kg) for antidiarrheal evaluation, and glibenclamide (10 mg/kg) for antidiabetic evaluation. Following a 12‐h fast for metabolic investigations, all medications were given orally by gavage using equal dosage volumes matched to body weight.

### 2.5 Analysis of In Vitro Antioxidant Activity

#### 2.5.1 DPPH Scavenging Assay

The antioxidant activity of the MECCB was assessed in vitro using the 2,2‐diphenyl‐1‐picrylhydrazyl (DPPH) radical scavenging test. A previously published method (Nur et al. 2023; Saraswati et al. 2020) was used as the basis for this procedure, with minimal modifications. MECCB and standard ascorbic acid were combined with a DPPH methanolic solution ranging from 31.25 to 500 μg/mL in a short amount of time and then allowed to incubate at room temperature for 30 min in the dark. Afterwards, a spectrophotometer (Thermo Scientific Evolution 200, USA) was used to measure the absorbance at 517 nm. The radical scavenging activity was calculated as [(Abs_control_ − Abs_sample_)/Abs_control_] × 100; the IC_50_ value was derived from the concentration‐response curve. The data were shown as the mean ± SEM, and each experiment was performed three times (*n* = 3).

### 2.6 Quantitative Phytochemical Examination

#### 2.6.1 Total Phenolic Content

The total phenolic content (TPC) of MECCB was determined using the Folin‐Ciocalteu method (Jung et al. 2008; Nur et al. 2023). The procedure consisted of mixing 10 mg of extract in 10 mL of methanol to prepare 1000 μg/mL solution. Serial dilutions of 1 mg/ml gallic acid solution were conducted to prepare 500, 250, 125, 62.5, and 31.25 μg/mL concentrations. To conduct the analysis, 1mL of the extract or different concentrations of gallic acid were combined with 2.5 mL of Folin‐Ciocalteu reagent (10 times diluted). Following 8 min at 25°C ± 2°C, 2.5 mL of 7.5% Na_2_CO₃ was introduced, and then 30 min of incubation at 40°C in the absence of light followed. Against a methanol blank, absorbance was recorded at 765 nm (Shimadzu UV‐1800). Calculated using a gallic acid standard curve (31.25–500 μg/mL, R2 > 0.99), TPC was expressed as mg GAE/g = (C × V)/M, where C is the gallic acid equivalent concentration (mg/mL) derived from the standard curve, V is the total volume of extract solution (mL), and M corresponds to the dry mass of extracted material (g). The mean ± SEM of all measurements was calculated using triplicate procedures.

#### 2.6.2 Total Flavonoid Content

The total flavonoid content (TFC) of the MECCB was measured using a modified aluminum chloride (AlCl3) colorimetric assay, with quercetin serving as the standard of measurement (Harun‐Or‐Rashid et al. 2023; Nur et al. 2023). A series of quercetin dilutions (31.25–500 μg/mL) was prepared, and the extract was obtained by mixing 10 mg of MECCB in 10 mL of methanol. 1 mL of the extract (1 mg/mL) or different concentrations of quercetin solutions was combined with 200 μL of aluminum chloride (10%), 200 μL of potassium acetate, and 5.6 mL of distilled water. After that, all of the test tubes were incubated at room temperature for 30 min, and the absorbance was subsequently measured at 506 nm. With C as the quercetin‐equivalent concentration, V as the extract volume, and M as the extracted mass, TFC was computed as TFC (mg QE/g) = (C × V/M). Every experiment was carried out in triplicate, and the results are presented as the mean ± SD.

#### 2.6.3 Total Condensed Tannin Assay

MECCB's overall condensed tannin (proanthocyanidin) content was calculated using a modified vanillin‐HCl assay (Kassim et al. 2011; Medini et al. 2014). A 0.5 mL solution of the extract (1 mg/mL) was briefly vortexed after being mixed with 3 mL of 4% vanillin in methanol (v/v) and 1.5 mL of strong hydrochloric acid. After being incubated at room temperature for 15 min, the absorbance was measured at 500 nm. The data were represented as milligrams of catechin equivalents (CE) per gram of extract, and a catechin standard calibration curve (0.1 mg/mL) was utilized for measurement. The data were displayed as the mean ± SEM, and all analyses were conducted in triplicate.

### 2.7 Antinociceptive Action

#### 2.7.1 Acetic Acid‐Induced Writhing Control Experiment

The writhing test was used to assess the peripheral antinociceptive activity of the methanolic extract of *C. castanocarpus* bark (MECCB) in *Swiss albino* mice (*n* = 3/group) (Ahmad et al. 2018; Ghosh et al. 2018). The mice were separated into four groups after a 3‐h fast. One group served as control, while the other received diclofenac sodium at a dose of 10 mg/kg i.p. The test groups received MECCB at doses of 200 and 400 mg/kg p.o. 30 min following treatment, 0.7% acetic acid (i.p.) was given to cause writhing, abdominal tightness, trunk twisting, and hind limb extension. Thirty minutes following the injection of acetic acid, the amount of writing was noted for 15 min. The percentage inhibition indicated as %Inhibition = [(Wcontrol − W treatment)/Wcontrol] × 100 was used to quantify the antinociceptive activity. Here, Wcontrol and Wtreatment represent the writhes in the negative control and treatment groups, respectively.

#### 2.7.2 Formal‐Induced Paw Licked Test

The formalin‐induced paw‐licking test was used to evaluate the central antinociceptive activity of the methanolic extract of *C. castanocarpus* bark (MECCB) in *Swiss albino* mice (*n* = 3 groups) (Estella et al. 2022; Jakaria et al. 2015). MECCB (200 and 400 mg/kg p.o.), standard medication (diclofenac sodium, 25 mg/kg i.p.), or vehicle (1% Tween‐80, 10 mL/kg p.o.) were administered to mice according to the same regimen as the writhing test. Twenty microliters of a formalin solution with a concentration of 2.5% were administered subcutaneously into the dorsal surface of the right hind paw using a needle 30 min after the treatment. Two phases of nociceptive behavior, licking and biting, were observed: early phase, 0–5 min post‐injection; late phase, 15–30 min post‐injection. Antinociceptive activity was estimated for each phase using the formula: % Inhibition = [(Lcontrol − Ltreatment)/Lcontrol] × 100, where Lcontrol and Ltreatment indicate the licking time (in seconds) for the control and treated groups, respectively.

### 2.8 In Vivo Antipyretic Activity

Brewer's yeast‐induced pyrexia in male and female *Swiss albino* mice (*n* = 3) was used to assess the antipyretic effect of *C. castanocarpus* bark methanolic extract (MECCB) (Emon, Alam, Rudra, Haidar, et al. 2021; Chattopadhyay et al. 2005). A significant fever response, peaking at 6–8 h and lasting up to 20 h, is produced by subcutaneous injection of a 20% yeast suspension, a common pyrogen used to induce fever in rodent models. This model is commonly used for studies on antipyretics (Wang et al. 2018). A 20% yeast suspension (10 mL/kg) administered subcutaneously caused fever following overnight fasting. The study comprised animals that showed a rectal temperature elevation of 0.3°C–0.5°C (measured digitally) 18 h after injection. Control groups received either 1% Tween‐80 (10 mL/kg p.o.), the standard group received indomethacin (4 mg/kg), and the test groups received MECCB (200 and 400 mg/kg p.o.). For 3 h after treatment, rectal temperatures were recorded hourly; each data point is the mean of 3 readings. To evaluate the efficacy of antipyretics, temperature fluctuations were calculated relative to baseline values.

### 2.9 In Vivo (Antidiarrheal) Action

#### 2.9.1 Castor Oil‐Induced Diarrhea

The antidiarrheal effect of *C. castanocarpus* bark methanolic extract (MECCB) was assessed using a Castor oil‐induced diarrhea model in *Swiss albino* mice (*n* = 3/group) (Alam et al. 2021; Gilbert et al. 2014). Animals were screened with 0.5 mL of castor oil (p.o.) following an 18‐h fast (water ad libitum), and only mice that exhibited diarrhea were chosen. The groups were given either (1) vehicle (1% Tween‐80 in water), (2) loperamide (5 mg/kg, positive control), or (3) MECCB (200 or 400 mg/kg). After treatment, 0.5 mL of castor oil was re‐administered one‐hour post‐treatment, and the mice were placed in absorbent paper‐covered cages. After recording 4 h of diarrheal episodes (wet/dry feces), the paper was changed (Jia et al. 2008). The calculation of antidiarrheal activity employed the following formula: % Inhibition = [(A − B)/A] × 100, where A and B indicate the mean quantity of diarrheal stools in the control group and the treatment group, respectively.

#### 2.9.2 Charcoal‐Marked Gastrointestinal Motility Test

A charcoal transit assay was used to assess gastrointestinal motility in fasting *Swiss albino* mice (*n* = 3 per group), using treatment protocols identical to those employed in the Castor oil‐induced diarrhea investigation (Ahmad et al. 2018; Shifah et al. 2020). An hour after being given a vehicle (1% Tween‐80), loperamide (5 mg/kg), or MECCB (200/400 mg/kg), mice were given 1 mL of a charcoal marker solution (10% activated charcoal in 5% gum acacia) orally. The animals were rendered unconscious 60 min after a chloroform overdose was administered, and the small intestine was promptly removed. The entire length of the digestive tract, as well as the distance traveled by the charcoal front from the pylorus to the cecum, were both measured. Charcoal was not used as a therapeutic agent; rather, it was used as a non‐absorbable marker to visualize gastrointestinal transit. The percentage inhibition of motility was determined by dividing the distance of charcoal migration (in cm) in the control group by the distance of migration (in cm) in the treatment group and then multiplying the result by 100. Furthermore, the peristalsis index was calculated as follows: (charcoal migration distance/total intestinal length) × 100.

### 2.10 Oral Glucose Tolerance Test (OGTT)

An oral glucose tolerance test was used to investigate the hypoglycemic activity of MECCB in *Swiss albino* mice that had been fasted for 12 h (*n* = 3 per group). The technique used for the test was developed, although some modifications were made to it (Derebe et al. 2020; Gobinath et al. 2022). The groups were given either (i) vehicle (1% Tween‐80 in water, negative control), (ii) glibenclamide (10 mg/kg, positive control), or (iii) MECCB (200 or 400 mg/kg). The 10% glucose solution was given during the fasting period before the hypoglycemic research to prevent dehydration and provide a source of calories (Sato et al. 2023; Zeleke et al. 2023). Thirty minutes following treatment, all animals were tested using a 10% glucose solution (2 g/kg p.o.). At baseline (zero minutes) and 30, 90, and 150 min after the glucose load, blood glucose levels were measured using a Bionime GS‐100 glucometer, which has an accuracy of 95%. The sampling was carried out through the tail vein. Total glycemic response analysis was performed using the area under the curve (AUC) calculations, and the results were presented in millimoles per liter (mmol/L).

### 2.11 Ethical Considerations

The procedures for conducting animal studies at the Department of Pharmacy, International Islamic University Chittagong, Bangladesh, were in full compliance with all applicable institutional rules. The study protocol received official clearance from the Institutional Animal Ethics Committee (Clearance Reference: IIUC/PHARM‐AEC‐74/10–19). The animals were cared for according to the most up‐to‐date standards for the use and treatment of laboratory animals throughout the study, with a focus on reducing their discomfort and pain.

### 2.12 Statistical Analysis

The findings of the experiments were presented as the mean ± SEM of three separate determinations, with three animals per group. For the statistical comparisons, we used GraphPad Prism 8.0 (GraphPad Software, USA) and ran a one‐way analysis of variance (ANOVA) followed by Dunnett's post hoc test. If the *p*‐value was less than 0.05, then the results were deemed to have statistical significance. Analyzed time‐course data, including glucose tolerance parameters, using a two‐way analysis of variance with Tukey's test, and dose‐dependent responses were investigated using the linear regression approach. When d*p* < 0.05, c*p* < 0.01, and a*p* < 0.0001, statistical analysis revealed a meaningful correlation.

## 3 Results

### 3.1 Evaluation of Antioxidant Action

**FIGURE 1:**
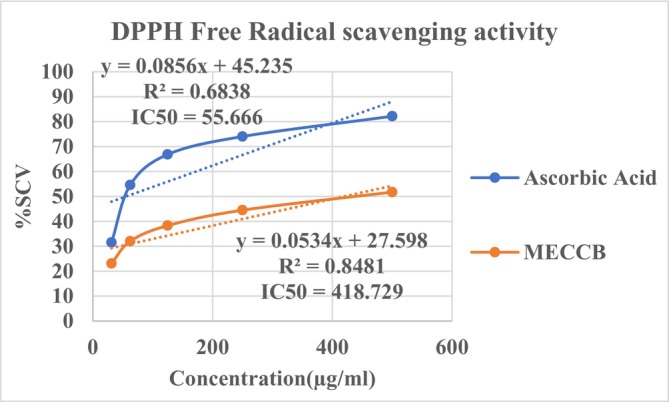
Linear regression curve of ascorbic acid and MECCB. MECCB = methanolic extract of *C. castanocarpus* bark.

The DPPH radical scavenging experiment was utilized in order to assess the antioxidant capacity of MECCB. Ascorbic acid served as the reference standard for this evaluation. Results from the dose–response curves showed that the regression equations for ascorbic acid were *y* = 0.0856*x* + 45.235 (*R* ^2^ = 0.6838), and for MECCB, they were *y* = 0.0534*x* + 27.598 (*R* ^2^ = 0.8481). The standard showed much stronger antioxidant activity, with IC50 values of 418.729 μg/mL for MECCB and 55.666 μg/mL for ascorbic acid, showing concentration‐dependent scavenging efficacy (Figure 1). These findings indicate that MECCB has a measurable capacity to scavenge free radicals; however, its potency is significantly lower than that of ascorbic acid.

### 3.2 Quantitative Phytochemical Content

**FIGURE 2:**
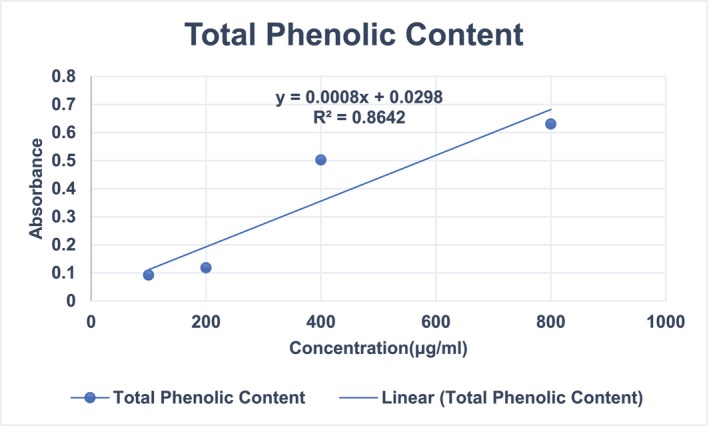
Linear regression calibration curve of Gallic acid for the determination of total phenolic content in MECCB. MECCB = methanolic extract of *C. castanocarpus* bark.

**FIGURE 3:**
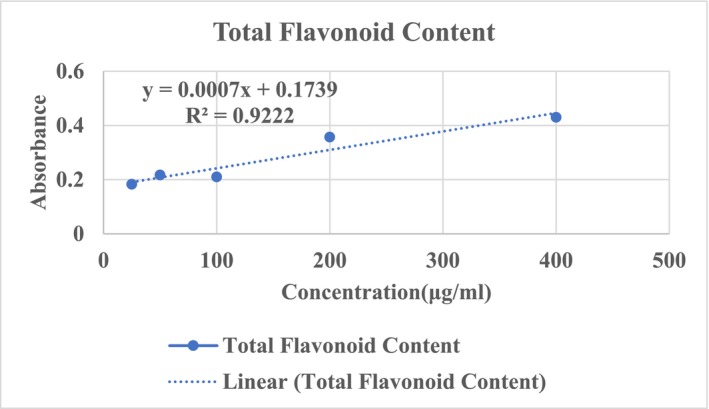
Linear regression calibration curve of quercetin determination of total flavonoid content in MECCB. MECCB = methanolic extract of *C. castanocarpus* bark.

**FIGURE 4:**
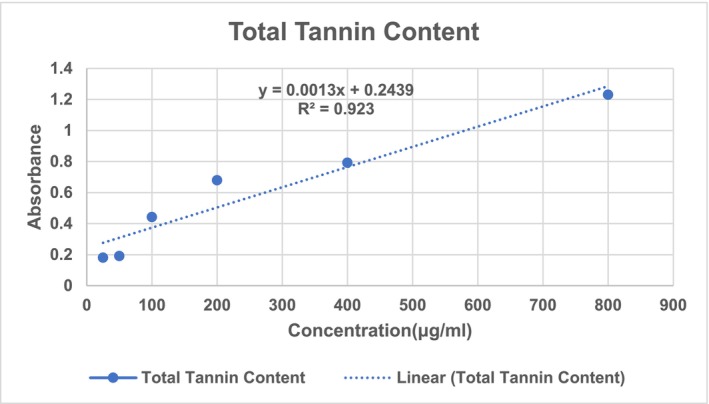
Linear regression calibration curve of catechin determination of total tannin content in MECCB. MECCB = methanolic extract of *C. castanocarpus* bark.

The MECCB extract included high concentrations of bioactive chemicals, as shown by the quantitative examination of phytochemical components. The total phenolic content (TPC) was evaluated by employing a gallic acid standard curve (*y* = 0.0008*x* + 0.0298; concentration range: 100–800 μg/mL) (Figure 2). The TPC was determined to be 364.9167 ± 27.0159 mg GAE/g D.E (Table 1). In a similar manner, the total flavonoid content (TFC) was determined to be 280.143 ± 45.122 mg QE/g D.E. (Table 1), which was quantified using a quercetin‐based regression equation (*y* = 0.0007*x* + 0.1739; range: 25–800 μg/mL) (Figure 3). As Table 1 summarizes, the total tannin content (TTC), measured using a catechin standard curve (*y* = 0.0013*x* + 0.2439; range: 25–800 μg/mL), yielded a comparable value of 351.4487 ± 13.239 mg CE/g D.E (Figure 4).

**TABLE 1:** Quantitative phytochemical content of MECCB.

Phytochemicals	Linear regression equation	Quantity
Total phenolic content	*y*= 0.0008*x* + 0.0298	369.9167 ± 27.0159 mg GAE/g
Total flavonoid content	*y* = 0.0007*x* + 0.1739	280.143 ± 45.122 mg QE/g
Total tannin content	*y*= 0.0013*x* + 0.2439	351.4467 ± 13.239 mg CE/g

### 3.3 Antinociceptive Activity

#### 3.3.1 The Acetic Acid‐Induced Writhing Inhibition Test

**FIGURE 5:**
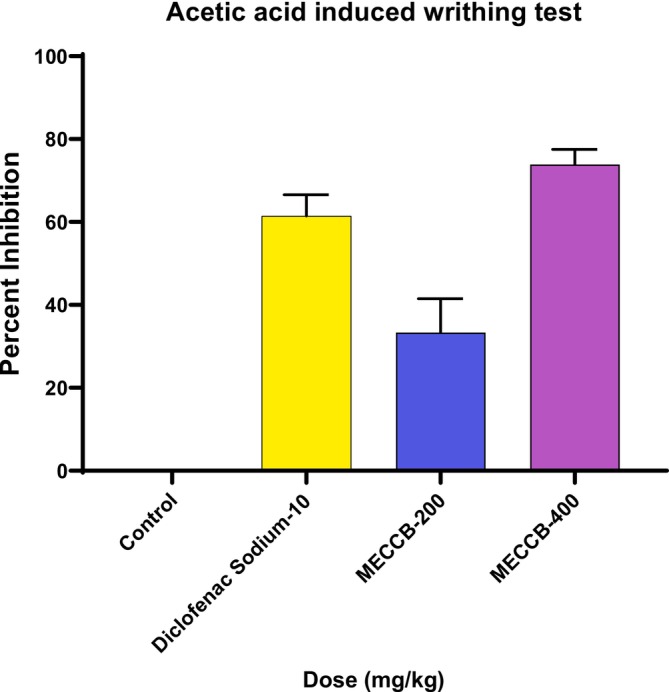
Values are mean ± SEM, (*n* = 3). A statistical representation of the percent inhibition of writhing by MECCB, standard (Diclofenac sodium, 10 mg/mL) processed by using GraphPad Prism for Windows, version 8.0. MECCB = methanolic extract of *C. castanocarpus* bark.

In the acetic acid‐induced writhing paradigm, the methanol extract of *Chaetocarpus castanocarpus* bark (MECCB) showed strong antinociceptive effects that were dosage dependent (Table 2, Figure 5). MECCB lowered the mean number of writhes to 34.00 ± 4.163 (*p* < 0.001) and 13.33 ± 1.856 (*p* < 0.001), respectively, at doses of 200 and 400 mg/kg against the control group (51.00 ± 2.9309). The highest dose (400 mg/kg) demonstrated 73.86% inhibition of writhing.

**TABLE 2:** Effect of MECCB on acetic acid‐induced writhing in mice.

Group	Dose (mg/kg‐B. w)	Frequency of writing (mean ± SEM)	% inhibition of analgesia
Control		51.00 ± 2.309	—
Diclofenac sodium	10	19.67 ± 2.603^b^	61.44%
MEBBF	200	34.00 ± 4.163^b^	33.33%
MEBBF	400	13.33 ± 1.856^a^	73.86%

*Note:* Values are presented as mean (% inhibition of analgesia) and mean ± SEM (frequency of writing) (*n* = 3). Superscript letters indicate significant differences vs. control (Tukey's test): a = *p* < 0.0001, b = *p* < 0.001, c = *p* < 0.01, d = *p* < 0.05.

#### 3.3.2 Formal Induced Writhing Inhibition Test

**FIGURE 6:**
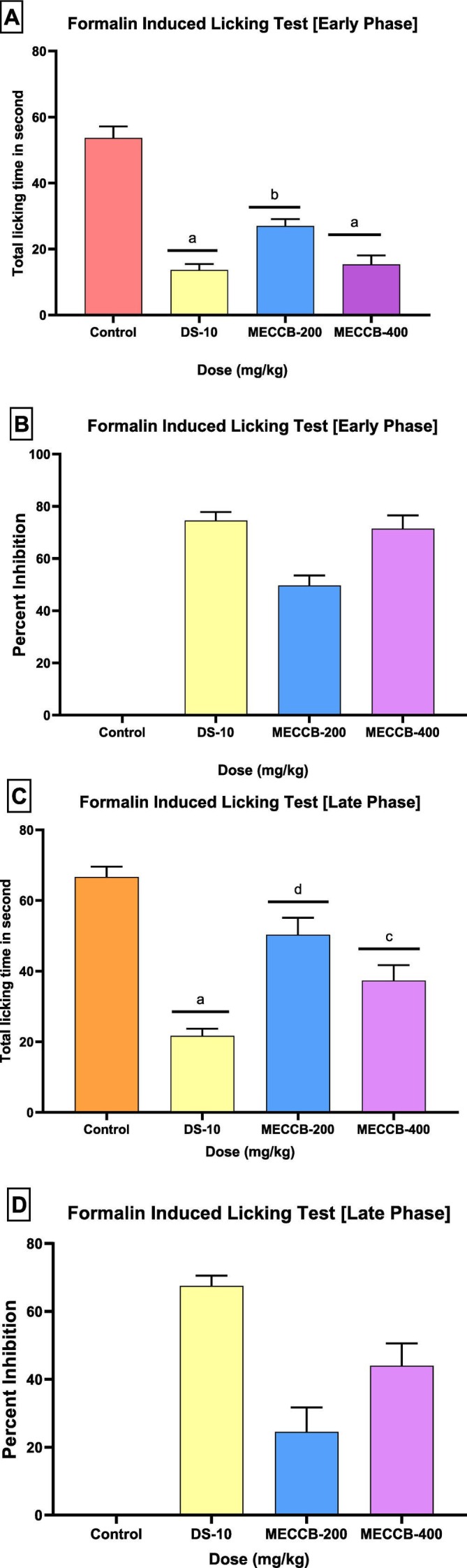
The frequency of writhing (A) and the percentage inhibition (B) during the early phase. The frequency of writhing (C) and the percentage inhibition (D) during the late phase. Values are mean ± SEM (*n* = 3); d = *p* < 0.05, c = *p* < 0.01, b = *p* < 0.001, and a = *p* < 0.0001, Dunnett's test as compared to control. A statistical representation of the frequency of writhing by MECCB, standard _ DS (Diclofenac sodium, 10 mg/mL) processed by (nonparametric or mixed)‐one way ANOVA analysis (Dunnett's test) using GraphPad Prism for Windows, version 8.0. MECCB = methanolic extract of *C. castanocarpus* bark.

Both the early (neurogenic) and late (inflammatory) phases of the formalin test showed that MECCB had strong central and peripheral antinociceptive activity (Figure 6A,B). Consistent with diclofenac sodium, a 400 mg/kg dose inhibited 71.43% of the early phase and 44.00% of the late phase, suggesting that both doses were equally effective. The extract's dual mode of action on pain pathways was confirmed when both the 200 and 400 mg/kg test doses caused dose‐dependent decreases in pain responses.

### 3.4 Yeast‐Caused Mice Pyrexia

**FIGURE 7:**
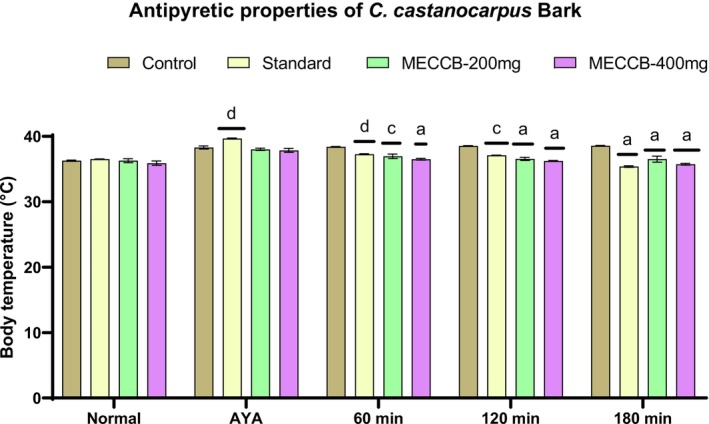
Results of antipyretic activity of MECCB. Values are mean ± SEM, (*n* = 3); d = *p* < 0.05, c = *p* < 0.01, b = *p* < 0.001, and a = *p* < 0.0001, Tukey's test as compared to control. A statistical representation of the body temperature reduced by MECCB, standard (Indomethacin, 4 mg/mL) processed by (nonparametric or mixed)‐two way ANOVA analysis (Tukey's test) using GraphPad Prism for Windows, version 8.0. MECCB = methanolic extract of *C. castanocarpus* bark.

In the brewer's yeast‐induced pyrexia model, the methanol extract of *Chaetocarpus castanocarpus* bark (MECCB) showed noticeable dose‐dependent antipyretic activity (Figure 7). After 18 h, control mice showed substantial hyperthermia following subcutaneous yeast injection; rectal temperatures rose from a baseline of 36.278°C ± 0.085°C to 38.556°C ± 0.056°C. MECCB treatment at 200 mg/kg significantly lowered the raised temperature to 36.519°C ± 0.455°C (*p* < 0.01), whereas the higher dose of 400 mg/kg demonstrated much better efficacy, dropping the temperature to 35.741°C ± 0.134°C (*p* < 0.001). These findings suggest that MECCB has strong fever‐reducing effects; the 400 mg/kg dosage shows the highest efficacy, which was maintained throughout the observation period. A one‐way ANOVA with Dunnett's test confirmed the significance of the data (*p* < 0.001 for 400 mg/kg dose versus yeast control), indicating MECCB's potential as an effective antipyretic drug.

### 3.5 Antidiarrheal Action

#### 3.5.1 Castor Oil‐Induced Mouse Diarrhea

**FIGURE 8:**
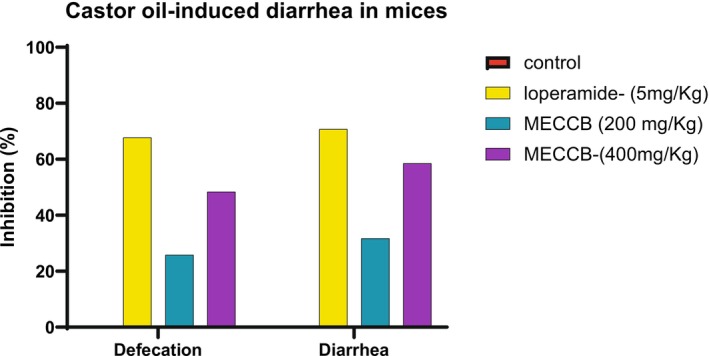
Inhibition of defection and diarrhea. Values are mean (*n* = 3). A statistical representation of the percent inhibition of defection and diarrhea by MECCB, standard (Loperamide 5 mg/kg) processed by using GraphPad Prism for Windows, version 8.0. MECCB = methanolic extract of *C. castanocarpus* bark.

In the Castor oil‐induced diarrhea method, the methanol extract of *Chaetocarpus castanocarpus* bark (MECCB) showed strong antidiarrheal activities. MECCB dosages of 200 and 400 mg/kg significantly reduced both total fecal discharge and phases of diarrhea in a dose‐dependent manner (Table 3, Figure 8). The higher dose (400 mg/kg) showed especially substantial efficacy, exhibiting 48.39% suppression of defecation (10.33 ± 1.45 feces vs. control 20.67 ± 0.33, *p* < 0.0001) and 58.53% inhibition of diarrhea feces (5.67 ± 0.88 vs. control 13.67 ± 1.20, *p* < 0.0001). Notably, these effects outperformed those of the standard medicine loperamide (5 mg/kg), which inhibited at 67.74% and 70.73%, respectively.

**TABLE 3:** Effect of MECCB on total and diarrheal feces count in castor oil‐induced diarrheal mice.

Treatment (mg/kg)	Total number of feces	% Inhibition of defecation	Total number of diarrheal feces	% Inhibition of defecation
Negative control (0.1 mL/mice)	20.67 ± o.33	—	13.67 ± 1.20	—
Loperamide‐5	6.667 ± 0.33^a^	67.74	4.0 ± 0.58^a^	70.73
MECCB‐200	15.33 ± 1.20^a^	25.81	9.343 ± 1.20^b^	31.70
MECCB‐400	10.33 ± 1.45^a^	48.39	5.67 ± 0.88^a^	58.53

*Note:* Values are presented as mean (% Inhibition of defecation) and mean ± SEM (Total number of diarrheal feces) (*n* = 3). Superscript letters indicate significant differences vs. control (Tukey's test): a = *p* < 0.0001, b = *p* < 0.001, c = *p* < 0.01, d = p < 0.05.

#### 3.5.2 Castor Oil Induced Intestinal Motility

**FIGURE 9:**
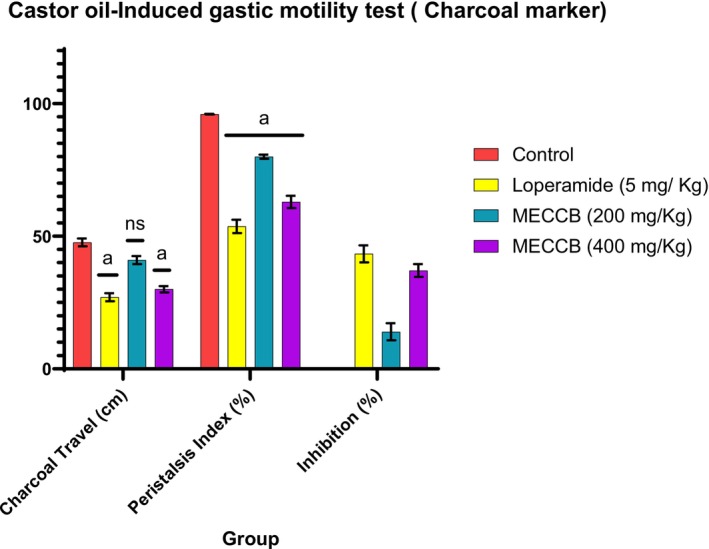
Results of the castor oil‐induced intestinal motility test. Values are mean ± SEM (*n* = 3); d = *p* < 0.05, c = *p* < 0.01, b = *p* < 0.001, and a = *p* < 0.0001, Dunnett's test as compared to control (Control (0.9% NaCl), 0.3 mL/mice). A statistical representation of the charcoal travel, peristalsis, and inhibition of gastric motility by MECCB, standard (Loperamide 5 mg/kg) processed by (nonparametric or mixed) one way ANOVA analysis (Dunnett's test) using GraphPad Prism for Windows, version 8.0. MECCB = methanolic extract of *C. castanocarpus* bark.

MECCB significantly reduced Castor oil‐induced intestinal hypermotility (Figure 9). The peristalsis index was reduced to 62.95 ± 2.31 (*p* < 0.001) by the 400 mg/kg dose, which is similar to loperamide's result (53.70 ± 2.52, *p* < 0.0001). At this dosage, the extract showed 37.06% ± 2.42% suppression of intestinal motility, almost matching the efficacy of loperamide (43.35% ± 3.21%).

### 3.6 Evaluation of Hypoglycemic Action

**FIGURE 10:**
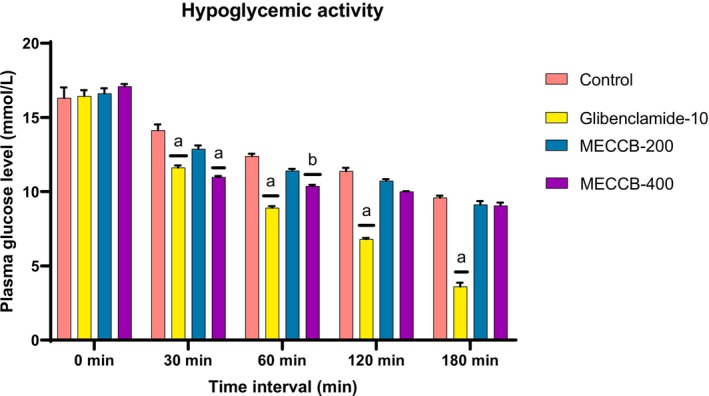
Results of the hypoglycemic activity of MECCB. Values are mean ± SEM, (*n* = 3); d = *p* < 0.05, c = *p* < 0.01, b = *p* < 0.001, and a = *p* < 0.0001, Tukey's test as compared to control (Control: 0.9% NaCl, 0.3 mL/mice). A statistical representation of the plasma glucose level reduced by MECCB, standard (Glibenclamide 10 mg/kg) processed by (nonparametric or mixed)‐one way ANOVA analysis (Dunnett's test) using GraphPad Prism for Windows, version 8.0. MECCB = methanolic extract of *C. castanocarpus* bark.

The oral glucose tolerance test (OGTT) revealed notable, dose‐dependent hypoglycemic effects from *C. castanocarpus* bark (MECCB) (Figure 10). Statistical analysis showed significant time effects (*F* (4,40) = 633, *p* < 0.001) and treatment effects (*F* (3,40) = 147, *p* < 0.001), along with a noteworthy factor interaction (*F* (12,40) = 22.5, *p* < 0.001). Blood glucose levels were shown to be considerably lower at 30 min (13.22 ± 0.42 vs. control 14.13 ± 0.24 mmol/L, *p* < 0.01), 60 min (11.41 ± 0.15 vs. 12.39 ± 0.19 mmol/L, *p* < 0.01), and 120 min (10.72 ± 0.16 vs. 11.38 ± 0.22 mmol/L, *p* < 0.05) when exposure to 200 mg/kg MECCB was seen. At 30 min (11.31 ± 0.38 mmol/L, *p* < 0.001), the larger 400 mg/kg dose showed more noticeable effects, attaining glucose reductions equivalent to glibenclamide (10 mg/kg). In the first hour after injection, both glibenclamide and MECCB 400 mg/kg resulted in comparable maximum glucose reductions (Δ = 6.5 mmol/L and Δ = 7.2 mmol/L, respectively). However, glibenclamide demonstrated better‐sustained activity at subsequent time periods (120 min: 6.79 ± 0.09 vs. MECCB 400 mg/kg 10.02 ± 0.04 mmol/L, *p* < 0.001). These results show that MECCB has strong antihyperglycemic effects, especially in acute glucose control, implying its possible use as a therapeutic agent for the control of postprandial hyperglycemia.

## 4 Discussion

Numerous studies have investigated medicinal plants and natural compounds with thrombolytic properties, with evidence suggesting preventive effects against cardiovascular events and stroke (Dwivedi et al. 2022; Islam et al. 2017). The bark extract of *Chaetocarpus castanocarpus* contained many bioactive components, such as tannins, terpenoids, alkaloids, glycosides, flavonoids, phenols, and coumarins, according to research conducted by (Rahman et al. 2024). By combining in vitro and in vivo methods, this study thoroughly evaluated the medicinal potential of methanol extract from *C. castanocarpus* bark (MECCB). The multi‐faceted medicinal effects of these phytoconstituents, including their antioxidant, analgesic, and anti‐inflammatory capabilities, have been extensively studied and documented (Asao and Asaduzzaman 2018). The present research demonstrates that MECCB exhibits a range of phytochemically‐based antinociceptive, antidiarrheal, antipyretic, hypoglycemic, and antioxidant effects. The results of this study are consistent with the traditional use of the plant and offer a scientific foundation for the prospective medicinal applications of the plant.

The high levels of TPC and TFC were supported by the antioxidant profile, which showed potent free radical scavenging (DPPH IC_50_ = 418.729 μg/mL). However, they exhibit a higher level of activity, which may be the result of synergistic interactions between the numerous phenolic components present in our extract. The extract's high total phenolic and flavonoid concentrations emphasize its antioxidant capacity, which is essential for reducing oxidative stress‐related diseases, including diabetes and inflammation (Medini et al. 2014; Moriasi et al. 2021).

The observed antinociceptive effects of MECCB in both formalin‐induced paw‐licking tests and acetic acid‐induced writhing suggest that it may modulate both peripheral and central pain pathways in this study. The significant antinociceptive effect observed in both the acetic acid‐induced writhing test (73.86% inhibition at 400 mg/kg) and the formalin test (71.43% and 44.00% inhibition in the early and late phases, respectively) suggests that the pain pathways in the body are being modulated. MECCB demonstrated more activity than diclofenac sodium (64.44% inhibition). The presence of tannins and alkaloids in the extract lends credence to the idea that the suppression of prostaglandin synthesis is the mechanism responsible for the substantial decrease in writhing and licking reactions, which is comparable to that of the conventional medication diclofenac sodium (Boarescu et al. 2023; Ghosh et al. 2018). This is especially important because traditional non‐steroidal anti‐inflammatory drugs have drawbacks, like gastrointestinal problems, that highlight the necessity for safer substitutes (Moriasi et al. 2021).

The antipyretic effect of MECCB (400 mg·kg^−1^) was particularly notable, as it surpassed that of indomethacin, effectively reducing yeast‐induced pyrexia. The multimodal activity of the extract on both prostaglandin and cytokine pathways (tumor necrosis factor alpha, interleukin‐6) may be the source of its great potency (Ilmi et al. 2021; Timbadiya et al. 2015). The antipyretic effects of the extract's flavonoids and alkaloids may be responsible for this effect (Khattak et al. 1985; Moriasi et al. 2021). This fits the growing curiosity about plant‐based antipyretics as substitutes for synthetic medications, which can have negative consequences.

MECCB exhibited antidiarrheal activity in both castor oil‐induced diarrhea and gastrointestinal motility tests. The results, showing a 58.53% suppression of diarrheal feces at 400 mg/kg and a 37.06% reduction in intestinal motility, are higher than those previously reported for a medicinal plant by (Alam et al. 2021), indicating that it may be helpful as a complete antidiarrheal drug. The extract presumably inhibited ricinoleic acid‐stimulated inflammatory mediators, including prostaglandins and nitric oxide, since it considerably decreased diarrheal episodes and intestinal motility (Gilbert et al. 2014; Shifah et al. 2020). Flavonoids, which are known to regulate intestinal secretion and motility, are present in MECCB at 280.143 ± 45.122 QE/g, which provides additional evidence of their activity. These results demonstrate the potential of MECCB as a natural remedy for diarrhea, particularly in regions with limited access to traditional antidiarrheal medications.

MECCB produced significant hypoglycemic effects in the oral glucose tolerance test, lowering blood glucose levels in a dose‐dependent manner. Compared to glibenclamide, MECCB has a faster onset and significantly lowers glucose levels in glucose tolerance tests. At 200 mg/kg, a noticeable decrease is observed after 30 min, and at 400 mg/kg, a significant decrease in glucose levels is noted (Δ = 6.5 mmol/L). The remarkable phenolic concentration of MECCB (364.917 ± 27.0159 mg GAE/g) is probably responsible for its superior performance compared to other studies on related species (Akter et al. 2023). Antidiabetic chemicals derived from plants often work by increasing glucose consumption or decreasing intestinal glucose absorption, which could explain this action (Derebe et al. 2020; Gobinath et al. 2022). Due to its flavonoid and polyphenol content, which enhances insulin sensitivity and mitigates oxidative stress, the extract possesses the characteristics of a natural antidiabetic (Akter et al. 2023).

This research provides compelling evidence that MECCB exhibits a wide range of pharmacological activities, which is supported by its extensive phytochemical profile. The results highlight its possibility as a natural therapeutic agent for oxidative stress, diabetes, pain, diarrhea, and fever. The incorporation of such plant‐based therapies into modern medicine may provide safer and more accessible therapeutic choices, particularly in resource‐limited areas.

### 4.1 Limitations of the Study

In this work, we acknowledge several limitations. The limited sample size employed for in vivo trials (*n* = 3 per group) is one of the study's limitations. Although this pilot design follows the 3Rs approach (Replacement, Reduction, Refinement) for initial activity screening, it diminishes statistical power and increases Type II errors. As a result, the effect sizes reported in this study should be considered preliminary, and the generalizability of the results is limited. Confirmatory studies with larger sample sizes (*n* ≥ 8 per group) are needed to validate the observed pharmacological effects and draw meaningful statistical inferences. Our preliminary analysis revealed no sex‐related differences, but the sample size was insufficient for a thorough sex‐disaggregated analysis. Future research with sufficient power per gender is necessary to corroborate this observation. A poor model fit is shown by the low *R* ^2^ value (0.76), which limits the accuracy of the IC_00_ estimate. As a result, these findings are only considered qualitative evidence. For a more robust *R* ^2^ value, future studies should use a larger concentration range and a four‐parameter logistic model. The DPPH assay, which provides little information across the full range of antioxidant activity, was the only method used for antioxidant assessment. Due to the inability to isolate or identify the precise bioactive chemicals causing the observed effects, phytochemical analysis was quantitative rather than qualitative. Underlying mechanisms of action, such as the participation of opioid receptors or inflammatory mediators, were not examined in this study. Despite being supported by the literature and based on preliminary range‐finding from an acute toxicity study conducted in our earlier work, the dose selection lacked a formal pharmacokinetic explanation. Thus, these findings should be viewed as preliminary, and to confirm and expand upon them, additional research is required using bigger samples, thorough phytochemical profiling, mechanistic investigations, and toxicity evaluations.

## 5 Conclusions and Recommendations

The findings of the current investigation indicate that plant extracts exhibit significant antinociceptive and antidiarrheal effects due to the presence of potentially bioactive compounds. Notably, MECCB demonstrated potent antipyretic activity in yeast‐induced pyrexia. In particular, the extract's hypoglycemic effects were noteworthy, as it showed a glibenclamide‐like fast glucose‐lowering action within 30 min. The presence of bioactive chemicals such as phenolics, flavonoids, and tannins is most likely responsible for these pharmacological effects. Despite its limitations, this investigation indicates that MECCB, an extract from *C. castanocarpus* bark, has pharmacological promise. As a crucial next step, advanced chromatographic profiling (GC–MS/MS) is used to link particular chemicals to pharmacological effects. To more thoroughly assess the extract's antioxidant activity, future research should use ABTS, FRAP, and cellular‐based models (e.g., cellular antioxidant activity assays). Additional molecular research is needed to clarify the processes behind its hypoglycemic and antinociceptive properties. To confirm effectiveness in humans, clinical trials are necessary; investigations into formulation may improve bioavailability. It may be possible to improve treatment results by investigating synergistic effects with standard pharmaceuticals. These actions will link evidence‐based applications with conventional understanding so enhancing MECCB's potential as a natural medicinal agent.

### 5.1 Author Agreement Statement

Esteemed Editor, this manuscript is original, has never been published before, and is not being considered for publication anywhere. All of the listed authors have reviewed and given their approval to the work, and we can confirm that no other individuals met the requirements for authorship but were not included in the list. In addition, we can state with confidence that everyone involved has given their stamp of approval to the manuscript's specified author order. We acknowledge that the Editorial process has one point of contact: the Corresponding Author. She is responsible for informing the other authors about the progress, revision submissions, and final approval of the proofs.

## Author Contributions

**Rakibur Rahman:** conceptualization, methodology, investigation, formal analysis, visualization, and original draft writing. **Farjana Akter Nipa:** investigation. **Taimun Siddique:** plant collection, extraction, and methodology. **Tania Rahman:** investigation and resources. **Abed Foyez Rafi:** investigation and resources. **Alfesani:** investigation and resources. **Ziaul Karim:** investigation and resources. **Tuhin Chandra Das:** investigation and resources. **Riniara Khatun:** conceptualization, supervision, and validation. **Abdur Rauf:** writing review and editing. **Hassan A. Hemeg:** writing review and editing.

## Data Availability

Data will be made available on request.
